# Beyond MD17: the reactive xxMD dataset

**DOI:** 10.1038/s41597-024-03019-3

**Published:** 2024-02-20

**Authors:** Zihan Pengmei, Junyu Liu, Yinan Shu

**Affiliations:** 1 Department of Chemistry, The University of Chicago, Chicago, IL 60637 USA; 2 Pritzker School of Molecular Engineering, The University of Chicago, Chicago, IL 60637 USA; 3 Department of Computer Science, The University of Chicago, Chicago, IL 60637 USA; 4 Kadanoff Center for Theoretical Physics, The University of Chicago, Chicago, IL 60637 USA; 5 qBraid Co., Chicago, IL 60615 USA; 6 SeQure, Chicago, IL 60615 USA; 7 Department of Chemistry, University of Minnesota, Minneapolis, MN 55414 USA

**Keywords:** Theoretical chemistry, Physical chemistry

## Abstract

System specific neural force fields (NFFs) have gained popularity in computational chemistry. One of the most popular datasets as a bencharmk to develop NFF models is the MD17 dataset and its subsequent extension. These datasets comprise geometries from the equilibrium region of the ground electronic state potential energy surface, sampled from direct adiabatic dynamics. However, many chemical reactions involve significant molecular geometrical deformations, for example, bond breaking. Therefore, MD17 is inadequate to represent a chemical reaction. To address this limitation in MD17, we introduce a new dataset, called Extended Excited-state Molecular Dynamics (xxMD) dataset. The xxMD dataset involves geometries sampled from direct nonadiabatic dynamics, and the energies are computed at both multireference wavefunction theory and density functional theory. We show that the xxMD dataset involves diverse geometries which represent chemical reactions. Assessment of NFF models on xxMD dataset reveals significantly higher predictive errors than those reported for MD17 and its variants. This work underscores the challenges faced in crafting a generalizable NFF model with extrapolation capability.

## Background & Summary

### Introduction

The development of molecular force fields driven by data is predominantly benchmarked against the MD17 dataset introduced by Chmiela *et al*.^1^ and its extension, the rMD17 dataset^2^. These datasets consist dynamic data of ten small to medium-sized gas-phase molecules. In molecular dynamics, data are intrinsically time-series sequences, necessitating careful sampling to prevent unintended information leakage into future states. A detailed analysis of MD17 and its variants reveals a significant sampling bias towards a narrow potential energy surface (PES) region close to the equilibrium structure. This narrow exploration of PES leads to limited conformation and energy space sampling, as our internal coordinate analysis shows. Thus, these datasets are suboptimal in terms of segmentation strategy and the molecular conformation space they cover.

**Fig. 1:**
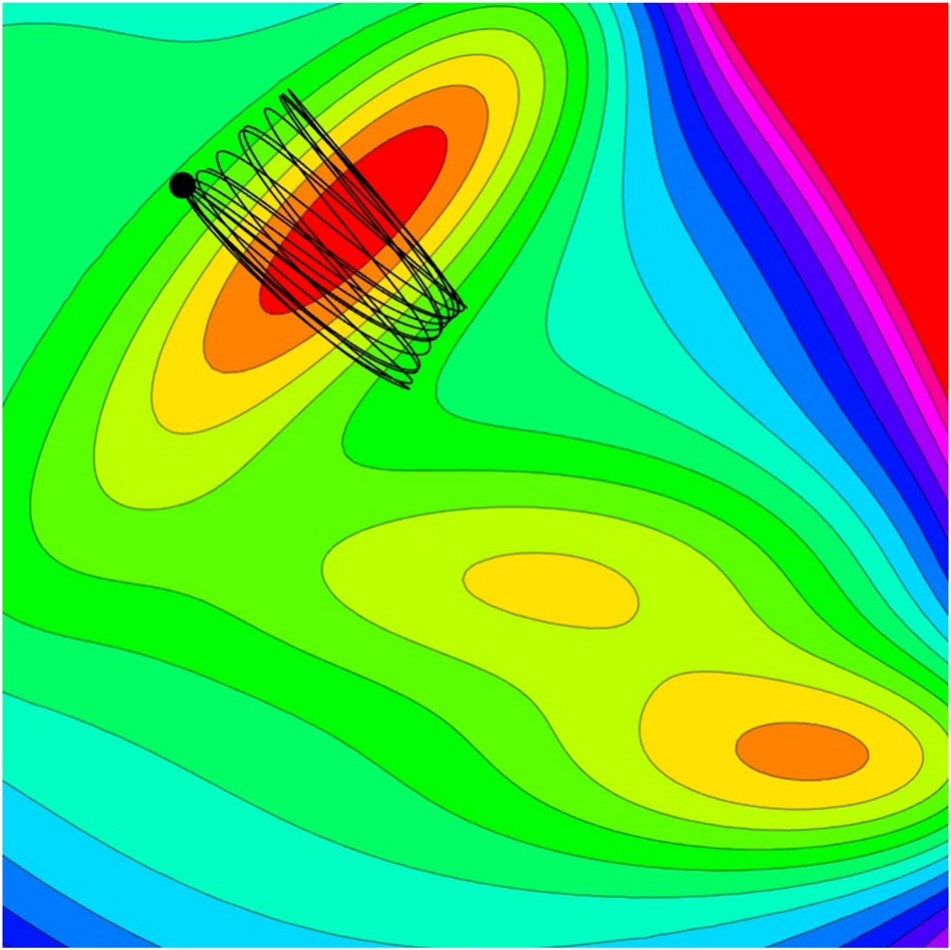
Trajectories on a representative potential energy surface. The contour plot represents the energy landscape, with the color gradient indicating various energy levels. Trajectories are usually confined to regions near the minima, reflecting the system’s preference for low-energy states close to or at equilibrium.

For our discussion, we refer to these conventional molecular dynamics datasets as in-distribution datasets. Yet, many chemical processes of interest occur out-of-distribution. Consider a basic chemical reaction depicted in Fig. 1: the nuclear configuration space includes reactants, transition states, and products. Sampling exclusively from the reactant region fails to capture the full dynamics of chemical reactions. As a result, NFF models trained on such skewed datasets are biased towards reactant configurations, potentially leading to qualitatively inaccurate predictions for a complete chemical reaction.

To overcome these challenges, we introduce the extended excited-state molecular dynamics (xxMD) dataset in this work. The xxMD retains the core objective of capturing trajectory data for small to medium-sized gas-phase molecules but distinguishes itself by incorporating nonadiabatic trajectories which include the dynamics of excited electronic states. Comprising four photochemically active molecules, the xxMD begins with significantly higher initial energies, enabling it to traverse a more extensive nuclear configuration space and more authentically represent the entire chemical reaction PES — reactants, transition states, and products. Notably, the xxMD captures regions near conical intersections, which are critical to the pathways of potential energy surfaces across different electronic states^3–8^. By including these key regions, the xxMD dataset aims to establish new benchmarks and challenges for NFF models, providing a more comprehensive and chemically accurate dataset for the development of predictive models.

We note that our development of xxMD datasets is not the first attempt ever to try to go beyond the (r)MD17 datasets. For example, the recently developed WS22 database^9^ tries to include nuclear configurations from multiple minima and interpolate among these configurations. Although WS22 has gone beyond (r)MD17, the xxMD datasets developed in current work involve much more complex configurations, for example, regions that correspond to conical intersections and locally avoided crossings.

### Existing datasets: MD17 and its variant

Chmiela *et al*. performed adiabatic ab initio molecular dynamics (AIMD) simulations on small gas-phase molecules at room temperature, with the electronic potential energies computed at the Kohn-Sham density functional theory (KS-DFT) level^1^. However, the original publication did not provide detailed specifics about the density functional, basis set, spin-polarization, grid for integration, and the software used. This lack of transparency presents a challenge for reproducibility and may limit the utility of the dataset for certain types of chemical simulation. Addressing the need for clarity, Christensen *et al*. revisited the  geometries of the MD17 dataset, recalculating them using the PBE density functional with the def2SVP basis set and enhanced grid precision^2^. This effort led to the creation of the rMD17 dataset, which has since been widely adopted in NFF studies^10,11^. Nonetheless, it is crucial to note the limitations of the PBE functional and def2SVP basis set for simulating accurate chemical reactions. While these computational tools can produce a continuous PES that varies with nuclear configuration, their ability to yield accurate results for chemically complex reactions — especially those involving bond breaking and formation — is often questioned. Despite these concerns, the MD17 and its refined counterpart, rMD17, are still considered to be well-behaved datasets for benchmarking purposes within certain constraints.

Adiabatic molecular dynamics datasets generated at low energy range are inherently limited in their sampling diversity and may not benefit fully from techniques such as uniform sampling and cross-validation. This is particularly true for adiabatic AIMD simulations, where initial low-energy conditions substantially constrain the nuclear configuration space. This limitation results in trajectories that predominantly occupy the reactant region of the PES, as depicted in Fig. 1.

**Fig. 2:**
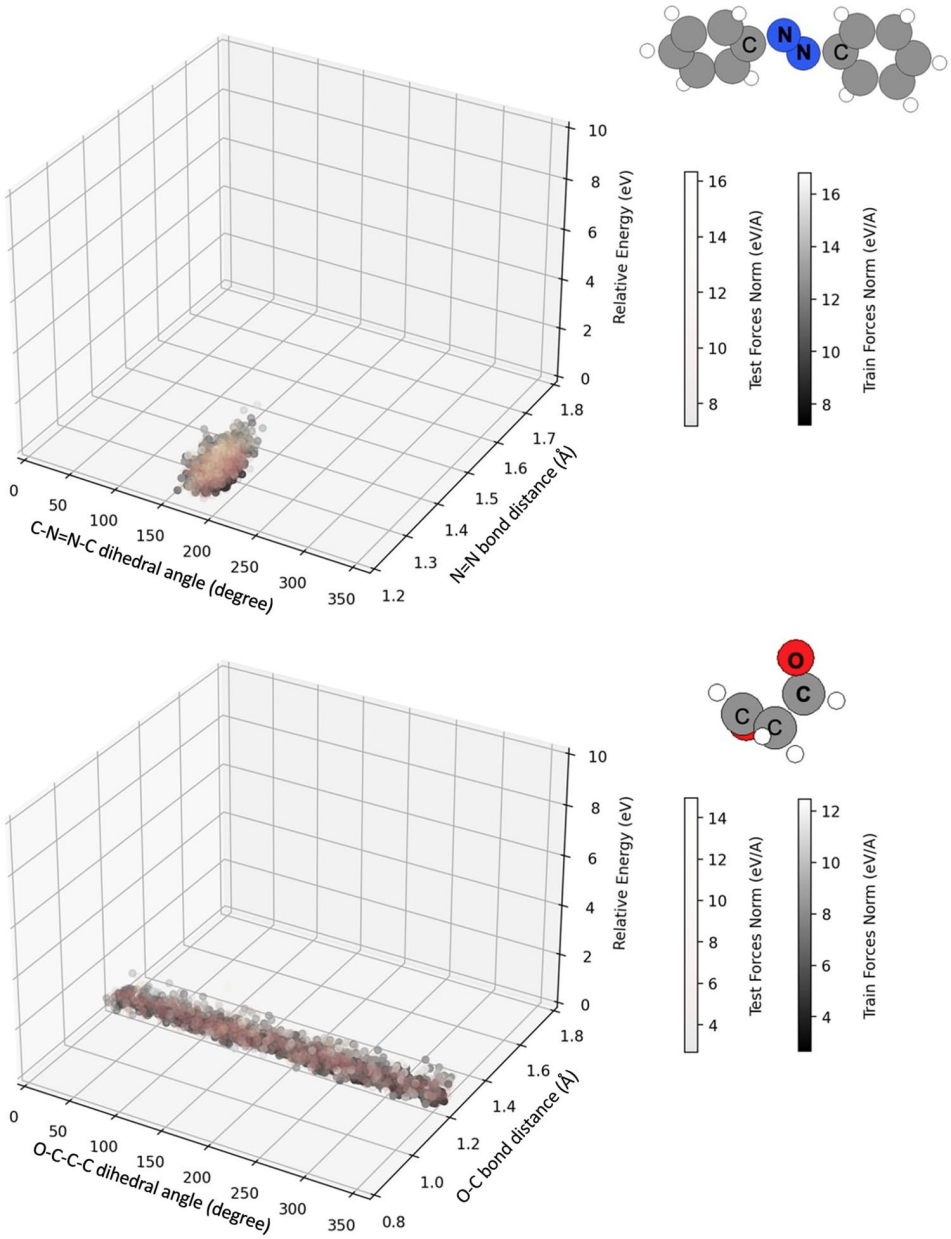
Illustration of training and testing sets using the reference split indices for azobenzene and malonaldehyde datasets in rMD17. The X-axis depicts dihedral angles (marked by ‘C’, ‘N’, and ‘O’), the Y-axis denotes bond distances (highlighted by bold letters), and the Z-axis shows relative energy. Training and testing samples are differentiated by color, correlating to force norms. Note that training samples overlap with testing ones.

To evaluate the breadth of configurations in the MD17 and rMD17 datasets, we conducted an analysis focused on internal coordinate distributions for azobenzene (C-N = N-C dihedral angle and the N = N bond length) and malonaldehyde (C-C-C = O dihedral angle and the C = O bond length). These distributions, along with the corresponding relative electronic potential energies and force norms, are illustrated in Fig. 2. The visual representation confirms that the internal coordinates distribution is notably narrow. Consequently, we observe a significant overlap between the training and testing samples within these datasets. Such overlap raises concerns about potential data leakage, which could inadvertently lead to overly optimistic results in benchmarking studies, as discussed in the literature^10–14^. The findings underscore the need for datasets that encompass a more diverse and extensive sampling of the PES to ensure robust and reliable benchmarks for NFF models.

### Dataset requirement

In classical MD and adiabatic AIMD simulations, chemical reactions are characterized by the system’s transition across different minima on the PES. These transitions correspond to changes in electronic potential energy as the system moves through various nuclear configurations. Systems naturally tend to follow the path of least resistance, referred to as the reaction pathway. To develop accurate NFFs, two fundamental elements are required: a comprehensive quantum chemical dataset that captures the full range of molecular transformations from various regions, and an advanced machine learning model with the capacity to interpolate and extrapolate across the PES. Fig. 1 illustrates typical low energy adiabatic AIMD trajectories on a PES. It’s evident that these low energy adiabatic AIMD trajectories tend to be localized around the ground state minima.

In contrast, datasets derived from nonadiabatic dynamics simulations are particularly valuable as they provide a more diverse array of nuclear configurations, going beyond the limitations of low energy adiabatic AIMD. These enriched datasets allow for the exploration of PES regions that are critical for understanding complex chemical processes, which are often not adequately represented in low energy adiabatic simulations.

### Summary

In summary, the xxMD dataset developed in current work includes four molecular systems: azobenzene, malonaldehyde, stilbene, and dithiophene, with crucial geometries along their reaction pathways illustrated in Figure S3. Notably, azobenzene and malonaldehyde are also part of the MD17 and rMD17 datasets, allowing for direct comparison.

The geometries in xxMD dataset are sampled from nonadiabatic dynamics. The potential energies and gradients, i.e. forces, for the first three singlet electronic states at the state-averaged complete active state self-consistent field (SA-CASSCF) level of theory^15^ are included in xxMD-CASSCF dataset. In addition, spin-polarized KS-DFT with M06 functional^16^ calculations are performed on the same geometries as in xxMD-CASSCF dataset, the resulting ground singlet electronic state potential energies and gradients are included in the xxMD-DFT dataset. Therefore, the xxMD datasets developed in current work involve a multi-state dataset — xxMD-CASSCF dataset, and a single-state dataset — xxMD-DFT dataset.

## Method

For our xxMD dataset, we employ the trajectory surface hopping (TSH) semiclassical nonadiabatic dynamics algorithm^3,4,17^ with SA-CASSCF electronic theory^15^. The SA-CASSCF is a multireference electronic structure theory that provides qualitatively correct description of strong correlation - which are critical for deformed geometries and conical intersections, while the linear response time dependent Kohn-Sham density function approximations failed qualitatively^18,19^. We ensured that only energy-conserving trajectories were sampled. The size of the data samples is detailed in Table S6 in supplementary material.

Nevertheless, to ensure compatibility with prevalent datasets like MD17, we also computed single-point spin-polarized KS-DFT (also called unrestricted KS-DFT) values. These calculations employ the M06^16^ exchange-correlation functional — a notably superior meta-GGA functional relative to PBE for chemical reactions. This dual approach culminates in two datasets: xxMD-CASSCF and xxMD-DFT. The former captures potential energies and forces across the first three electronic states for azobenzene, dithiophene, malonaldehyde, and stilbene. The latter provides recomputed ground-state energy and force values, anchored on the same trajectories. All computational details are described in supplementary information section G Computational details. Notice that SA-CASSCF PESs can be more complicated than DFT surfaces due to more complicated electronic structure algorithm from SA-CASSCF, i.e. choice of active space. Both xxMD datasets are structured via a temporal split method, partitioning training and testing data based on trajectory timesteps. We want to emphasize that xxMD datasets do not involve nonadiabatic coupling vectors (NACs) for two reasons: first, the advances in the field of nonadiabatic dynamics have enabled NAC-free nonadiabatic dynamics simulations, for example, curvature-driven dynamics^20–24^. Second, the purpose of the current work is to provide a database which includes a wide nuclear configuration space for which the energies and gradients of multiple electronic states are available. Therefore, the machine learning force field models can be tested against each surfaces. We note that an appropriate fit of a coupled PESs with multiple electronic states for a single system requires diabatic representation, which is beyond the discussion of the current work^25–27^.

We evaluated six message-passing NFF models on the xxMD datasets: SchNet^28^, DimeNet++ (DPP)^29^, SphereNet (SPN)^14^, NequIP^10^, Allegro^30^, and MACE^11^. Each model was mostly used with its default parameters, and in line with convention, we trained the NFFs emphasizing more on force losses. While hyperparameter optimization could potentially improve performance (See Supplementary Information for an example), it remains outside the scope of this study. Therefore, the presented results might not showcase the absolute best performance for each model. Given our observations, we encourage researchers aiming to apply NFFs in practical scenarios to conduct rigorous re-benchmarks tailored to their specific chemical systems and objectives.

**Fig. 3:**
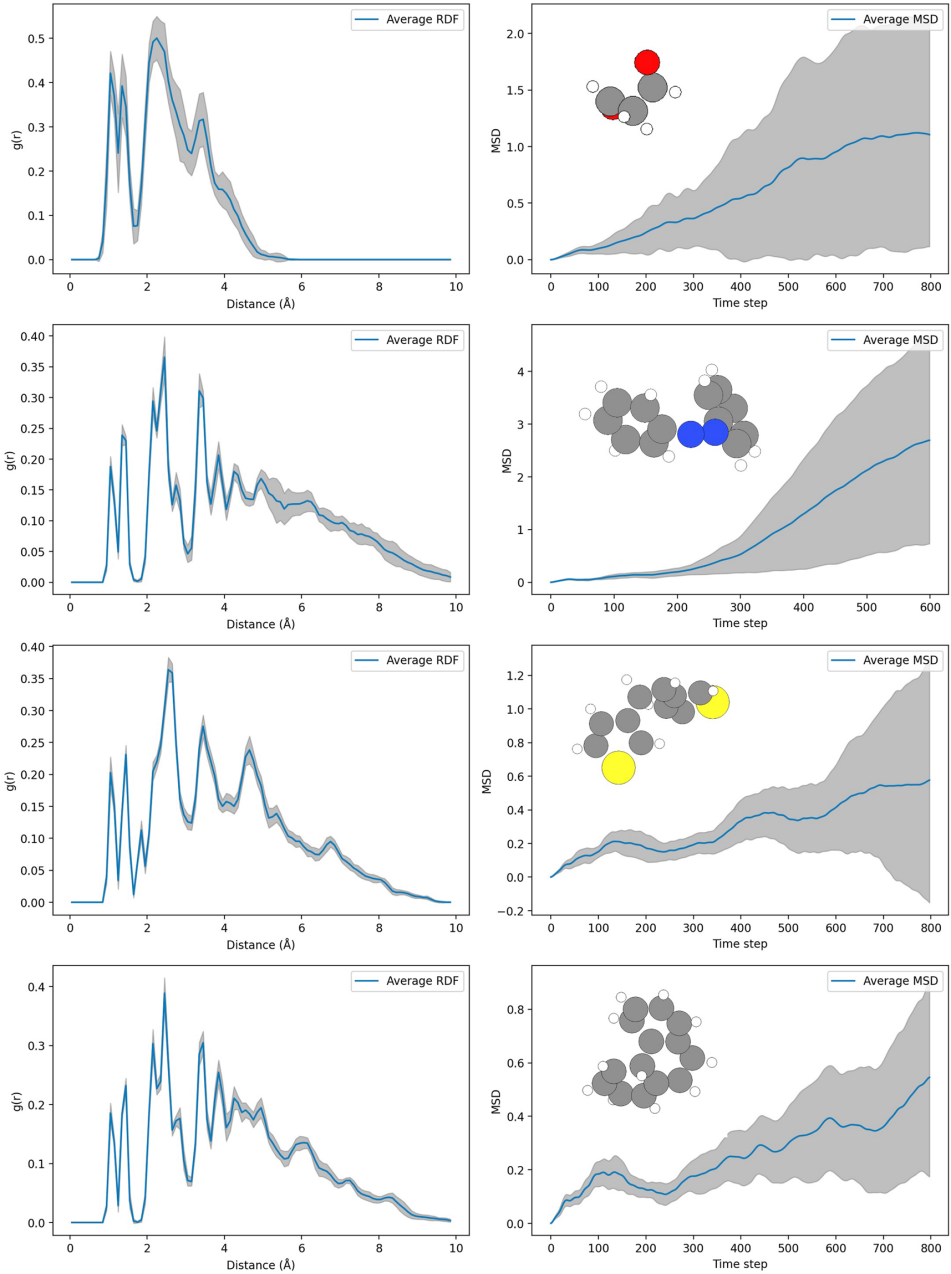
Comparison of Average RDFs and MSDs Across Multiple Trajectories. Each row corresponds to a group of trajectories, with RDF on the left (indicating particle density as a function of distance) and MSD on the right (showing particle displacement over time). Shaded regions represent standard deviations.

Temporal splitting was chosen over random splitting to partition the xxMD datasets. This method involves dividing time-series data based on timesteps, reserving a specific range for testing and applying a 50:25:25 split for training, validation, and testing sets. Such a split allows for a rigorous assessment of a model’s ability to predict unexplored areas of the PES. This is highlighted in Fig. 3, where deviations in trajectories over time emphasize the datasets’ capability to challenge and evaluate the extrapolative power of NFFs. However, it is possible to use random splitting on xxMD datasets considering the wide coverage of conformation space.

## Data Records

The xxMD-CASSCF and xxMD-DFT datasets have been made publicly available on GitHub at the following URL: https://github.com/zpengmei/xxMD; and on Zenodo at the following URL: 10.5281/zenodo.10393859^31^. These datasets are stored in compressed archives, each containing pre-split extended XYZ format files based on temporal information. The files have been processed using the Atomic Simulation Environment (ASE) software package, as documented in the reference^32^. The GitHub repository is structured into two main directories, each corresponding to one of the datasets: xxMD-CASSCF and xxMD-DFT.

Within each directory, data is further organized into subdirectories named after the four molecules studied: malonaldehyde, azobenzene, stilbene, and dithiophene. Each molecule’s subdirectory contains the associated dataset files. Notably, the xxMD-CASSCF dataset includes an additional subdirectory structure that segregates the state-specific data for the first three electronic states.

## Technical Validation

### Dynamic properties

Through the ensemble-averaged radial distribution function (RDF) and mean square displacement (MSD), the xxMD datasets exhibit a comprehensive sampling of the nuclear configuration space, surpassing that observed in MD17. Illustrated in Fig. 3, the RDF and MSD track nuclear configurations over time, offering insights into the spatial distribution and mobility of particles, respectively. The RDF measures the likelihood of particle presence at varying radial distances from a reference point, whereas the MSD quantifies the average squared distance that molecules travel over a time interval.

The pronounced shifts in nuclear configurations captured by nonadiabatic dynamics in the xxMD datasets, as reflected in the dynamic breadth of the RDF and MSD, underline the enhanced diversity of PES regions sampled. Consequently, the complexity of mastering the PESs for molecules in the xxMD dataset is expected to be significantly elevated, presenting a robust challenge for the accuracy of NFFs.

### Benchmarks on xxMD-CASSCF and xxMD-DFT datasets

We picked six representative equivariant NFFs to benchmark. The hyperparameters and training details of models are described in the supplementary information. We used a weighted loss of 1:1000 on energy and forces. We stress that our purpose is not to perform an extensive comparison of models over multiple choices of hyperparameters. Rather, we limit ourselves to showing the performance of the models in the default configurations.

We first evaluate the regression precision of all models on the first three electronic states, which are labeled as S_0_, S_1_, and S_2_ respectively (Label S denotes the singlet spin state which is a widely used notation in quantum chemistry) by using the temporal splitting approach for data in xxMD-CASSCF dataset. The mean absolute error (MAE) of the predictive energies and forces for test sets are shown in Table 1. Similarly, we present such results of using xxMD-DFT datasets in Table 2. The best performance on each row is bolded. Additional results on the validation sets are available in the supporting information. Note that validation sets depict the nuclear configurations that are closer to the training sets due to the temporal splitting. Therefore, the MAE shown in validation sets are in general lower than that for test sets.

**Table 1:** Comparison of predictive MAE of energy(E, meV) and forces(F, meV/A) on hold-out testing set for different models on temporally split xxMD-CASSCF datasets and tasks.

Dataset	State	Task	MACE	Allegro	NequIP	SchNet	DPP	SPN
Azobenzene	S_0_	E	527	**437**	870	648	528	493
		F	**63**	82	76	156	102	96
	S_1_	E	599	524	1160	619	497	**494**
		F	**78**	98	85	157	91	88
	S_2_	E	881	783	1957	894	837	**831**
		F	**191**	216	215	284	224	231
Dithiophene	S_0_	E	293	296	295	306	295	**290**
		F	**14**	31	21	94	30	31
	S_1_	E	205	211	224	217	**204**	205
		F	**37**	81	49	103	41	44
	S_2_	E	246	255	259	262	**244**	246
		F	52	10	70	121	**51**	54
Malonaldehyde	S_0_	E	530	443	770	515	452	**442**
		F	**105**	142	166	220	138	137
	S_1_	E	528	**458**	1227	482	482	462
		F	164	189	189	260	165	**161**
	S_2_	E	679	**528**	159	653	610	615
		F	276	307	309	353	251	**238**
Stilbene	S_0_	E	538	544	529	604	**519**	544
		F	**72**	87	112	191	91	114
	S_1_	E	391	353	370	424	**313**	352
		F	**58**	66	85	142	88	93
	S_2_	E	604	669	674	678	550	**529**
		F	**117**	142	178	259	148	159

**Table 2:** Comparison of predictive MAE of energy(E, meV) and forces(F, meV/A) on hold-out testing set for different models xxMD-DFT datasets and tasks with temporal split.

Dataset	Task	MACE	Allegro	NequIP	SchNet	DPP	SPN
Azobenzene	E	292	**174**	1754	722	300	260
	F	**85**	110	129	283	173	168
Stilbene	E	**315**	332	647	397	439	477
	F	**149**	189	156	291	162	168
Malonaldehyde	E	190	**151**	244	360	179	185
	F	**166**	210	227	394	257	255
Dithiophene	E	100	103	243	323	**61**	76
	F	**51**	75	101	177	74	90

### Comparison with existing datasets

In this section, we analyze model behavior for two molecules, namely azobenzene and malonaldehyde. These two molecules are both available in xxMD and (r)MD17 datasets. Benchmarks for (r)MD17 reveal that the accuracy of MACE, NequIP, and SPN exceeds that of traditional electronic structure methods^10,11,14,33^. It’s essential to note that typical errors for KS-DFT in predicting relative transition state energy can be several kcal/mol. For instance, the MAEs of HTBH38 (Hydrogen transfer barrier heights) and NHTBH38 (non-Hydrogen transfer barrier heights) databases are about 9.1 kcal/mol for PBE and 2.4 kcal/mol for M06. Thus, an NFF fitting error below 50 meV would surpass the accuracy of modern density functional calculations. However, such claims are pertinent mainly to ground state potential energies, given that excited state calculations are often less precise. Therefore, given the reported MAEs, these NFF models perform admirably on (r)MD17 datasets.

However, this conclusion might be deceiving. Previous discussions highlight the constrained nuclear configuration space in MD17 and rMD17. A comparative analysis of MAEs for the six NFF models on azobenzene and malonaldehyde from xxMD-DFT and (r)MD17 is presented in Table 3. Literature-derived MD17/rMD17 results indicate that all models used 1,000 training samples^10,11,14^. Predictably, the predictive prowess of NFF models diminishes when applied to the xxMD dataset.

**Table 3:** Comparison of predictive MAE on hold-out testing sets of NFF models on azobenzene and malonaldehyde in (r)MD17 and xxMD-DFT datasets. (r)MD17 benchmarks with 1,000 samples are taken from^11,14,28^.

Molecule	Dataset	Task	MACE	Allegro	NequIP	SchNet	DPP	SPN
Azobenzene	rMD17	E	1.2	1.2	0.7	N/A	N/A	N/A
		F	3.0	2.6	2.9	N/A	N/A	N/A
	xxMD	E	292	174	1754	722	300	260
		F	85	110	129	283	173	168
Malonaldehyde	(r)MD17	E	0.8	0.6	0.8	5.6	4.5	N/A
		F	4.1	3.6	5.1	28.6	16.6	7.5
	xxMD	E	190	151	244	360	179	185
		F	166	210	227	394	257	255

The differences of MAEs for a same NFF model for rMD17 and xxMD come from two aspects, namely, the differences in dataset, and the differences in splitting method. The xxMD datasets contain much more complex nuclear configurations than (r)MD17. For the splitting method, one can have either random splitting or temporal splitting. For certain purposes, for example, if one uses the trajectory data to construct a global PES for the system, random splitting would be a good approach. For purpose of extended trajectory simulation with existing trajectory data, temporal splitting may be favored. Because the ultimate goal is to look for unknown chemical events that may not be observed from short trajectory simulations. In that spirit, we use temporal splitting in the current work. For the purpose of extended trajectory simulation, random splitting, which has been used to test against (r)MD17 dataset, means a severe leakage of future information. In practice, if we would like to model a chemical reaction, it would be impractical to manually sample every relevant region on the potential energy surfaces. Therefore, it is a desired property for an NFF model has the capability of physical extrapolation to some extent. Physical extrapolation is achieved in several models, for examples, reactive force field^34^, and use of a parametrically managed activation function^35^.

The effectiveness of NFF models largely depends on the datasets they are benchmarked against. Historically, the (r)MD17 datasets have been the gold standard for this purpose. However, our study highlights the potential shortcomings of relying solely on (r)MD17 datasets. Given that they primarily capture a narrow nuclear configuration space from low energy ground state AIMDs, they fall short of encompassing the holistic nuclear configuration pertinent to chemical reactions. Training NFF models on such datasets can be somewhat trivial and could result in misleading conclusions about their true capabilities. For instances, computational chemists have a long history of using system specific force fields, which can be easily developed by computing a hessian at the ground state equilibrium geometry^36,37^.

To address this gap, we introduced the xxMD dataset, derived from nonadiabatic dynamics trajectories. The xxMD dataset offers a comprehensive representation of the nuclear configuration space, encapsulating the reactant, transition state, product, and conical intersection regions of PESs. Its inclusion of several low-lying excited state potential energy surfaces underscores its importance and the challenges it presents for NFF model development. Our benchmarks of prevailing NFF models on the xxMD dataset have revealed pronounced difficulties. Utilizing default hyperparameters, the chosen NFF models struggled to offer quantitatively or even qualitatively accurate force field models for specific systems. We anticipate that our findings will galvanize the community towards pioneering more advanced NFF models better equipped to study intricate chemical reactions.

## Code availablity

Nonadiabatic dynamics are performed with Surface Hopping with Arbitrart Coupling (SHARC) code, which is available at https://github.com/sharc-md/sharc. SchNet, DimeNet++ and SphereNet are available as implemented in the Dive Into Graphs package (https://github.com/divelab/DIG.git). NequIP package is available at https://github.com/mir-group/nequip.git. Allegro package is available at https://github.com/mir-group/allegro. MACE package is available at https://github.com/ACEsuit/mace.git. All packages are up-to-date at the data of the publication. All the trainings are done with single precision float format. SchNet, DPP and SPN models are initialized using the default hyperparameters shipped with the packages. Allegro hyperameters can be found at https://github.com/mir-group/allegro/blob/main/configs/example.yaml, NequIP hyperparameters are available at https://github.com/mir-group/nequip/blob/main/configs/example.yaml, MACE hyperparameters are available at https://github.com/ACEsuit/mace. Since Dive Into Graphs package doesn’t implement the scale and shift of the energy, we manually rescaled the energy by substracting the energy of the configuration with the lowest potential energy.

### Supplementary information

Supplementary Information for

## References

[1] Chmiela S (2017). Machine learning of accurate energy-conserving molecular force fields. Science advances.

[2] Christensen AS, Von Lilienfeld OA (2020). On the role of gradients for machine learning of molecular energies and forces. Machine Learning: Science and Technology.

[3] Tully JC, Preston RK (1971). Trajectory surface hopping approach to nonadiabatic molecular collisions: The reaction of h+ with d2. The Journal of chemical physics.

[4] Blais NC, Truhlar DG (1983). Trajectory-surface-hopping study of Na(3*p*
^2^P) + H_2_ - > Na(3 s ^2^S) + H2(*v*’, *j*’, *θ*). The Journal of chemical physics.

[5] Herman MF (1984). Nonadiabatic semiclassical scattering. i. analysis of generalized surface hopping procedures. The Journal of chemical physics.

[6] Tully JC (1990). Molecular dynamics with electronic transitions. The Journal of Chemical Physics.

[7] Yarkony DR (1996). Diabaolical conical intersections. Reviews of Modern Physics.

[8] Levine BG (2019). Conical intersections at the nanoscale: Molecular ideas for materials. Annual Review of Physical Chemistry.

[9] Pinheiro M, Zhang S, Dral PO, Barbatti M (2023). Ws22 database, wigner sampling and geometry interpolation for configurationally diverse molecular datasets. Scientific Data.

[10] Batzner S (2022). E(3)-equivariant graph neural networks for data-efficient and accurate interatomic potentials. Nature communications.

[11] Batatia I, Kovacs DP, Simm G, Ortner C, Csányi G (2022). Mace: Higher order equivariant message passing neural networks for fast and accurate force fields. Advances in Neural Information Processing Systems.

[12] Batatia, I. *et al*. The design space of e (3)-equivariant atom-centered interatomic potentials. *arXiv preprint arXiv:2205.06643* (2022). 10.1038/s42256-024-00956-x PMC11769842 39877429

[13] Schütt, K., Unke, O. & Gastegger, M. Equivariant message passing for the prediction of tensorial properties and molecular spectra. In *International Conference on Machine Learning*, 9377–9388 (PMLR, 2021).

[14] Liu, Y. *et al*. Spherical message passing for 3d graph networks. *arXiv preprint arXiv:2102.05013* (2021).

[15] Roos BO, Taylor PR, Sigbahn PEM (1980). A complete active space scf method (casscf) using a density matrix formulated super-ci approach. Chemical Physics.

[16] Zhao Y, Truhlar DG (2008). The m06 suite of density functionals for main group thermochemistry, thermochemical kinetics, noncovalent interactions, excited states, and transition elements: two new functionals and systematic testing of four m06-class functionals and 12 other functionals. Theoretical chemistry accounts.

[17] Barbatti M (2011). Nonadiabatic dyanmics with trajectory surface hopping method. WIREs Computational Molecular Science.

[18] Levine BG, Ko C, Quenneville J, Martinez TJ (2006). Conical intersections and double excitations in time-dependent density functional theory. Molecular Physics.

[19] Shu Y, Parker KA, Truhlar DG (2017). Dual-functional tamm-dancoff approximation: A convenient density functional method that correctly describes s1/s0 conical intersections. Journal of Physical Chemistry Letters.

[20] Shu Y (2022). Dynamics algorithms with only potential energies and gradients: Curvature-driven coherent switching with decay of mixing and curvature-driven trajectory surface hopping. Journal of Chemical Theory and Computation.

[21] do Casal MT, Toldo MJ, Pinheiro M, Barbatti M (2021). Fewest switches surface hopping with baeck-an couplings. Open Research Europe.

[22] Zhang L (2022). Nonadiabatic dynamics of 1,3-cyclohexadiene by curvature-driven coherent switching with decay of mixing. Journal of Chemical Theory and Computation.

[23] Zhao X, Shu Y, Zhang L, Xu X, Truhlar DG (2023). Direct nonadiabatic dynamics of ammonia with curvature-driven coherent switching with decay of mixing and with fewest switches with time uncertainty: An illustration of population leaking in trajectory surface hopping due to frustrated hops. Journal of Chemical Theory and Computation.

[24] Zhao X (2023). Nonadiabatic coupling in trajectory surface hopping: Accurate time derivative coupling by the curvature-driven approximation. Journal of Chemical Theory and Computation.

[25] Shu Y, Truhlar DG (2020). Diabatization by machine intelligence. Journal of Chemical Theory and Computation.

[26] Shu Y, Varga Z, Sampaio de Oliveira-Filho AG, Truhlar DG (2021). Permutationally restrained diabatization by machine intelligence. Journal of Chemical Theory and Computation.

[27] Shu Y, Varga Z, Kanchanakungwankul S, Zhang L, Truhlar DG (2022). Diabatic states of molecules. Journal of Physical Chemistry A.

[28] Schütt, K. *et al*. Schnet: A continuous-filter convolutional neural network for modeling quantum interactions. *Advances in neural information processing systems* **30** (2017).

[29] Gasteiger, J., Giri, S., Margraf, J. T. & Günnemann, S. Fast and uncertainty-aware directional message passing for non-equilibrium molecules. *arXiv preprint arXiv:2011.14115* (2020).

[30] Musaelian A (2023). Learning local equivariant representations for large-scale atomistic dynamics. Nature Communications.

[31] Pengmei Z, Liu J, Shu Y (2023). reactive xxMD dataset..

[32] Larsen AH (2017). The atomic simulation environment—a python library for working with atoms. Journal of Physics: Condensed Matter.

[33] Mardirossian N, Head-Gordon M (2017). Thirty years of density functional theory in computational chemistry: an overview and extensive assessment of 200 density functionals. Molecular Physics.

[34] van Duin ACT, Dasgupta S, Lorant F, Goddard WA (2001). Reaxff: A reactive force field for hydrocarbons. Journal of Physical Chemistry A.

[35] Akher FB, Shu Y, Varga Z, Bhaumik S, Truhlar DG (2023). Parametrically managed activation function for fitting a neural network potential with physical behavior enforced by a low-dimensional potential. Journal of Physical Chemistry A.

[36] Cacelli I, Prampolini G (2007). Parametrization and validation of intramolecular force fields derived from dft calculations. Journal of Chemical Theory and Computation.

[37] Vanduyfhuys L (2016). Quickff: A program for a quick and easy derivation of force fields for metal-organic frameworks from ab initio input. Journal of Computational Chemistry.

