# Supplementary material for: Beyond MD17: the reactive xxMD dataset
Source: Sci Data. 2024 Feb 20;11:222. doi: 10.1038/s41597-024-03019-3 (PMC10879526; doi:10.1038/s41597-024-03019-3)
Supplement: Supplementary file 1 — Supplementary Information for [file 41597_2024_3019_MOESM1_ESM.pdf]

Supplementary Information Table of Contents

A Preliminaries of dynamics 1
B Brief introduction of chosen neural force fields 1
C Timing 2
D Additional illustration of xxMD datasets 3
E Benchmark results on the validation sets of xxMD-CASSCF and xxMD-DFT 5
F Addition experiment of hyperparameter tuning 5
G Computational details 5
G.1 Dynamics . . . 6
G.2 Complete Active Space Self-Consistent Field (CASSCF) . . . 7
G.3 Unrestricted KS-DFT . . . 8
G.4 Dihydrogen dissociation: a comparative case of RKS, UKS and CASSCF . . . 9
References 9

A Preliminaries of dynamics

In the realm of quantum mechanics, the behavior of nuclei is ideally described by the time-dependent Schrödinger equation. Yet, practical computation limits restrict nuclear quantum dynamics simulations to small systems with just 5 or 6 atoms. Consequently, in many cases, the nuclei are treated as classical particles. This premise paves the way for classical Molecular Dynamics (MD) and adiabatic Ab Initio Molecular Dynamics (AIMD), wherein the dynamics are propagated based on a **single electronic state**.

At the heart of classical MD is the Newtonian equation of motion:

$$m_i \frac{d^2\mathbf{r}_i}{dt^2} = \mathbf{F}_i \tag{1}$$

where m_i denotes the mass of atom i, $\mathbf{r}_i$ its position, and $\mathbf{F}_i$ the force exerted on it. This force can be described as the negative gradient of the potential energy V at the atom's location:

$$\mathbf{F}_i = -\nabla V(\mathbf{r}_i) \tag{2}$$

The ground state electronic potential energy, $V(\mathbf{r}_i)$, in the absence of an external field, forms the basis for the PES. Classical force fields offer an analytical approximation of this energy based on nuclear configuration:

$$V(\mathbf{r}) = V_{\text{bond}}(\mathbf{r}) + V_{\text{angle}}(\mathbf{r}) + V_{\text{dihedral}}(\mathbf{r}) + V_{\text{non-bonded}}(\mathbf{r}) \tag{3}$$

This classical approximation often falls short under quantum mechanical scenarios, particularly during bond breaks, necessitating improvements in force field formulations. Upon electronic excitation, as observed in solar cells or photochemical reactions, nuclei confront electronic potentials beyond the ground state. Herein, dynamics involving multiple electronic states emerge. Nonadiabatic dynamics, particularly pertinent when energy levels soar, may either adopt the trajectory surface hopping method or the semiclassical Ehrenfest dynamics, depending on the specific conditions.

B Brief introduction of chosen neural force fields

In this study, we picked six representative neural network architectures for NFF applications, namely, SchNet[1], DPP[2], SPN[3], NequIP[4], Allegro[5] and MACE[6]. In general, those approaches can be devided into two catagories based on the representation of the feature space. SchNet, DPP and SPN are the so-called scaler-based NFFs, while NequIP, MACE and Allegro are vector-based NFFs, as we summarized in Table S1.

The key concept in SchNet is the continuous-filter convolution, which involves two steps: interaction and update. In the interaction step, the model calculates pairwise interaction features between all atoms based on their distances, using a set of radial bessel basis. The update step then uses these interaction features to update the atom-centered descriptors. In DPP, a higher-order feature, bond angle has been introduced to enhance the expressiveness of the neural network. DPP uses a concept called spherical functions to account for the directionality of the interactions between atoms. The DPP architecture uses 'interaction blocks' to propagate information through the molecular graph. Each interaction block consists of a radial and a spherical part. The radial part captures the distance-based interactions, similar to SchNet. The spherical part captures the

angular interactions among any three atoms in the molecule, which is unique to DPP. As a continuation of the DPP, SPN further introduces another higher-order feature called dihedral angles among any four atoms in the molecule. These improvements are chemically-intuitive since bond lengths, angles, and dihedral angles are very common descriptors in classical force fields[7].

Table S1. Summary of models, their features, and the corresponding years of introduction.

Model	Feature	Year
SchNet	Bond length	2017
DPP	Bond length, Bond angle	2020
SPN	Bond length, Bond angle, Dihedral angle	2021
NequIP	SO(3) vector	2021
Allegro	SO(3) vector	2022
MACE	SO(3) vector	2022

On the other hand, NequIP, Allegro, and MACE are examples of group equivariant NFFs that based on SO(3) relative displacement vectors between any two atoms in the molecule. These networks use the representation theory of the three-dimensional orthogonal group to construct neurons that obey equivariance with respect rotations and reflections of a molecular system's pose. We visualize this concept in Figure S1. Atomic types are embedded as node features, and relative displacement vectors that contains the positional information are converted into activations that transform according to irreducible representations (irreps) of the orthogonal group. Nonlinearities for these activations are constructed using the tensor product, followed by applying the Clebsch-Gordon decomposition to convert the product back into irreducible components.

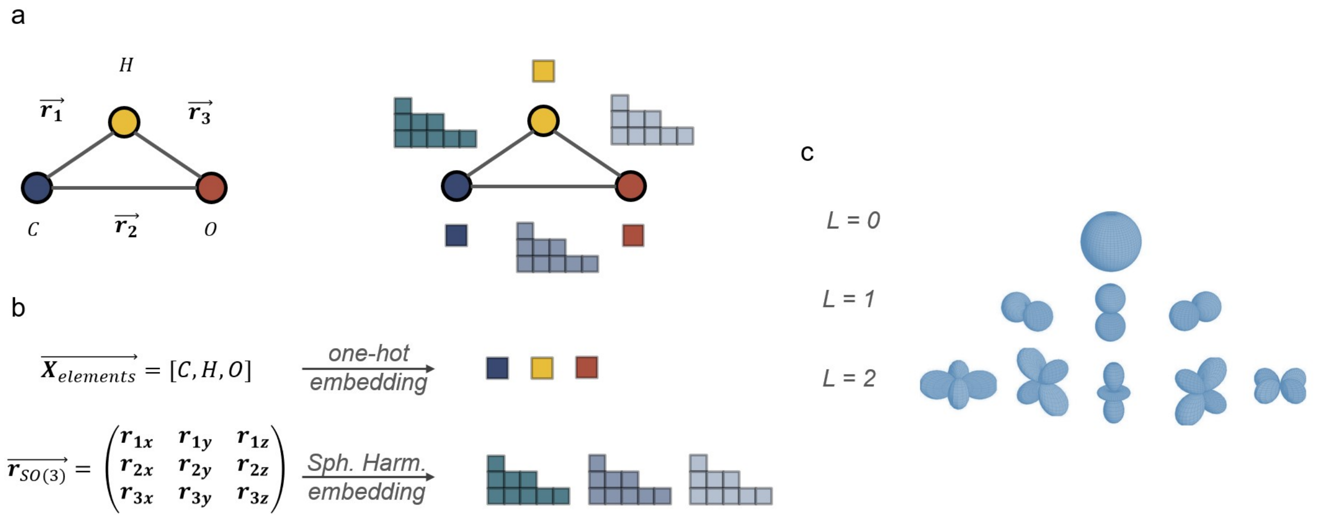

Figure S1. (a) depicts three atoms with their relative displacement vectors. (b) illustrates the details of atomic embedding with E(3) equivariant activations based on spherical harmonics and one-hot encoding for chemical elements. (c) gives an illustration of spherical harmonics with quantum numbers $L = 0, 1, 2$. The Clebsch-Gordan coefficients are used during the aggregation step to ensure rotational equivariance when combining activations with different irreps.

C Timing

In this practical view, we present a comprehensive analysis of the operational time of multiple NFFs examined in our study as illustrated in Figure S2. It is important to note that the specific runtime of each NFF model is contingent upon the chosen setup and hyperparameter selection. For example, the radius cutoff utilized for generating locally fully-connected graphs can yield varying numbers of edges and nodes in each mini-batch. Within our findings, we have diligently reported the time required for processing each sample in a mini-batch using the designated hyperparameters. Consequently, we emphasize that while we employed mostly default hyperparameters as a practical reference.

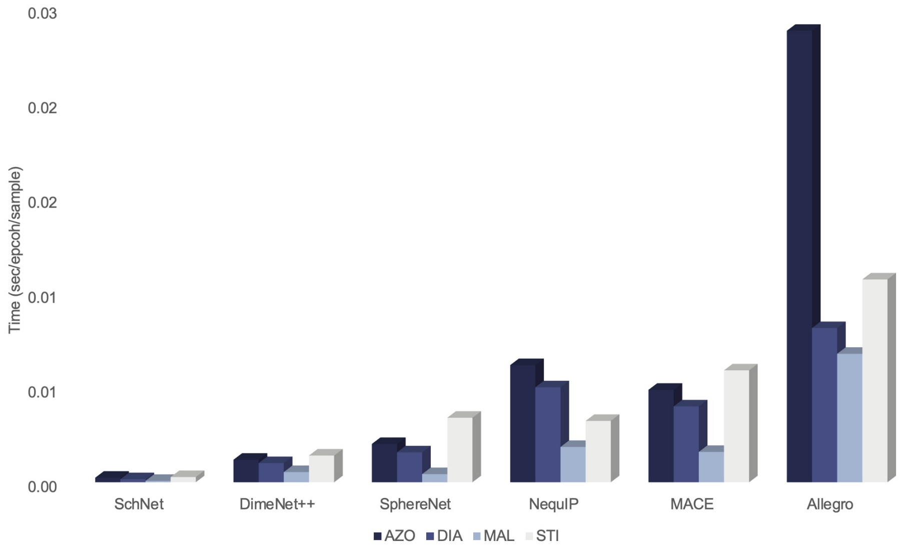

Figure S2. Comparison of average computational time for NFFs. The timing is specific to the chosen hyperparameters. All NFFs, except MACE, operate with single precision. Generally, group-equivariant NFFs are significantly more computationally expensive.

D Additional illustration of xxMD datasets

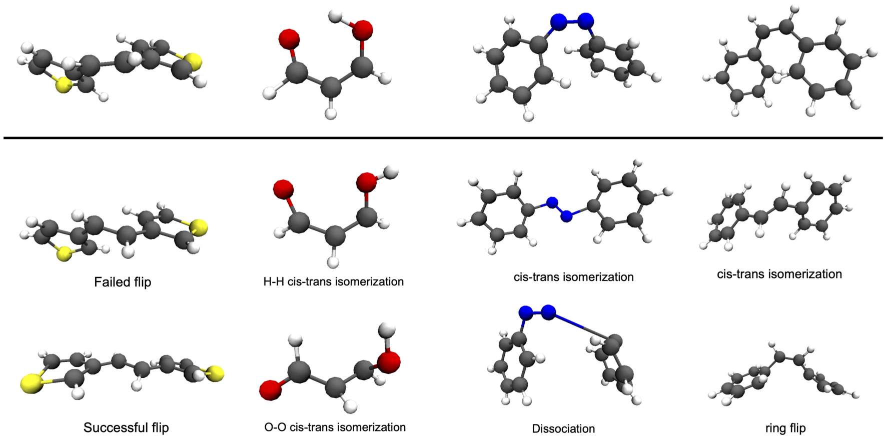

Figure S3. Schematic representation of the photodynamic processes featured in the xxMD dataset.

Here we provide addition illustration (Figure S4) of the xxMD-CASSCF datasets with the ground-state energy and forces as the internal coordinate analysis of MD17. For azobenzene, the primary reaction path involves the cis-trans isomerization of the two phenyl groups along the N=N bond. For malonaldehyde, the reaction path involves either a H-H cis-trans isomerization occurs along the O=C bond or a O-O cis-trans isomerization occurs along the carbon skeleton. The reaction path of stilbene involves the cis-trans isomerization of the two phenyl rings along the C=C double bond and the flip of the phenyl rings to opposite directions. The reaction path of dithiophene is also the cis-trans isomerization of two five-member rings along the C=C double bond.

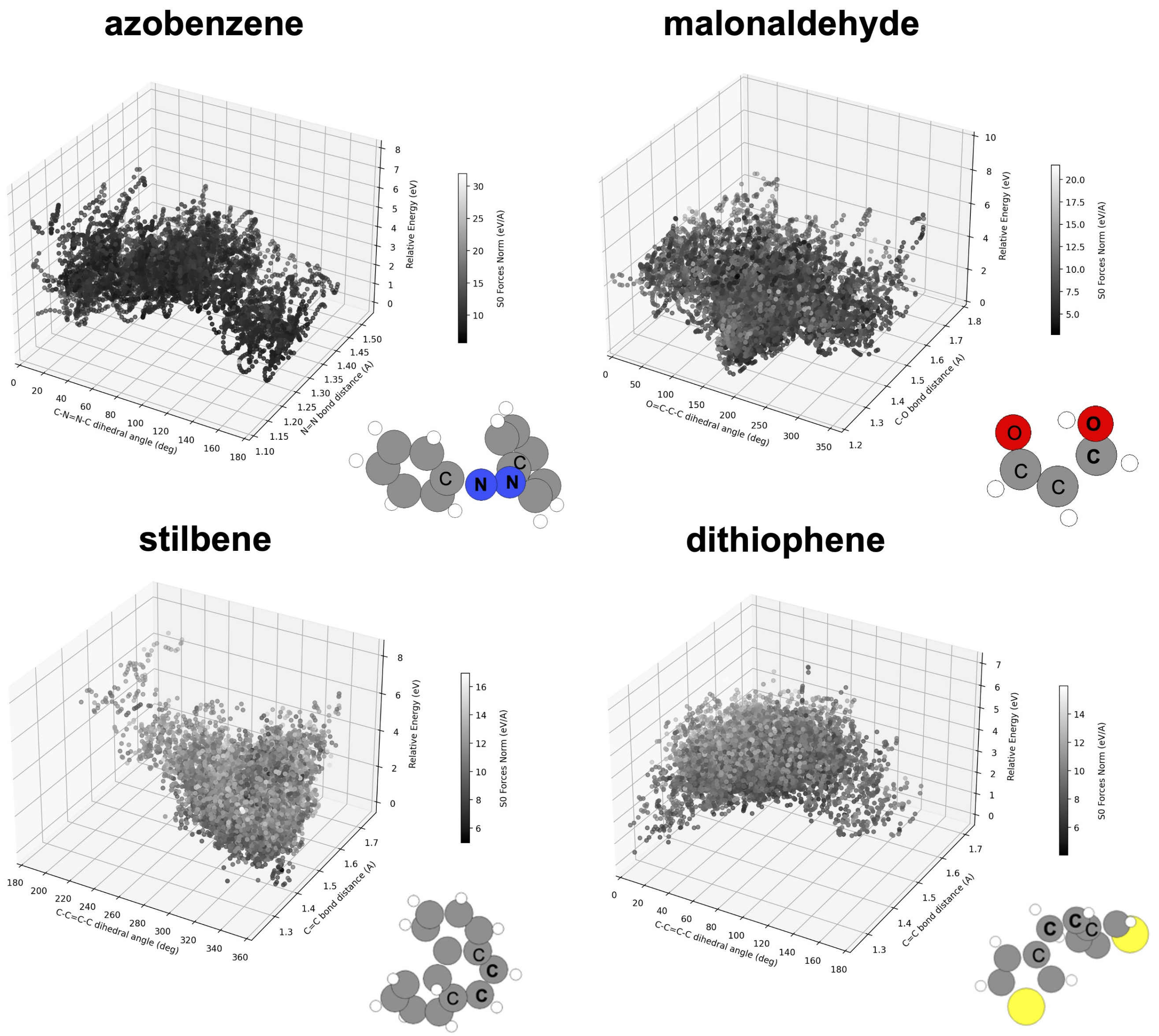

Figure S4. Illustration of xxMD datasets using similar internal coordinates as MD17 analysis. Only the ground-state (in black/white color scheme) energies are visualized for clarity. This figure clearly indicates the breadth of the conformation space explored by using direct non-adiabatic dynamics as compared to MD simulation with room temperature.

E Benchmark results on the validation sets of xxMD-CASSCF and xxMD-DFT

Table S2. Comparison of predictive MAE on validation set for different models on temporally split xxMD-CASSCF datasets and tasks. Energy(E) has the unit of meV, while forces(F) have the unit of meV/A.

Dataset	State	Task	MACE	Allegro	NequIP	SchNet	DPP	SPN
Azobenzene	S_0	E	527	**367**	530	682	526	494
		F	**50**	70	69	141	83	79
	S_1	E	474	**308**	869	483	478	428
		F	**67**	83	74	134	79	74
	S_2	E	864	**742**	1590	897	804	801
		F	**163**	185	180	257	191	185
Dithiophene	S_0	E	300	295	304	302	**286**	287
		F	**10**	21	17	76	22	24
	S_1	E	259	208	226	219	**206**	208
		F	65	78	46	101	**33**	36
	S_2	E	246	258	256	259	**244**	249
		F	50	104	69	119	**49**	51
Malonaldehyde	S_0	E	488	**386**	583	470	419	415
		F	**84**	147	109	179	108	109
	S_1	E	507	**406**	828	469	446	451
		F	**144**	184	168	233	147	145
	S_2	E	556	**457**	858	526	512	512
		F	221	255	281	301	197	**188**
Stilbene	S_0	E	517	514	**359**	505	467	461
		F	54	71	**12**	145	71	75
	S_1	E	322	293	**262**	351	294	316
		F	38	45	**20**	97	62	61
	S_2	E	494	505	**377**	596	486	473
		F	80	98	**31**	176	104	106

F Addition experiment of hyperparameter tuning

We would like to stress again, our purpose is to give a initial view of the datasets using common hyperparameters without tuning, and we don't aim to strictly test models listed. We left most hyperparameters unchanged as default, and uses a loss weight heavily focused on the forces following the literatures[4,6,8]. However, users should carefully use the hyperparameters before applying to specific chemical problems.

We used a default MACE model and one subset of xxMD-CASSCF dataset and varied the weights on the energy and forces, and we found that by simply tuning this hyperparameter, MACE would perform noticeably differently. For instance, the regression accuracy on force is not improved and accuracy on energy deteriorates quickly when the weight on the force gradually increase from 1 to 1000. On the contrary, putting slightly more weights on the energy greatly improve the overall performance, as we laid out in Table S4. Thus, we would like to leave a note to future users that exploring the hyperparameter spaces is important.

G Computational details

The active space and basis set used for SA-CASSCF for all four molecules are shwon in Table S5. The total number of trajectories simulated are vary, but finally selected number of points for each molecule in xxMD dataset is summarized in Table S6 as well. These points are selected from energy conserving trajectories only, and we used the criteria for the total energy conservation as listed in Table S6. Therefore, all trajectories fail to conserve the total energy below the threshold are discarded. We show the total energy conservation in Figure S5

Table S3. Comparison of predictive MAE on validation set for different models xxMD-DFT datasets and tasks with temporal split. Energy(E) has the unit of meV, while forces(F) have the unit of meV/A.

Dataset	Task	MACE	Allegro	NequIP	SchNet	DPP	SPN
Azobenzene	E	257	106	393	539	184	**168**
	F	**71**	98	119	248	150	140
Stilbene	E	190	200	161	**156**	224	248
	F	**104**	116	117	196	114	125
Malonaldehyde	E	156	**91**	134	257	116	127
	F	**135**	162	173	326	208	204
Dithiophene	E	89	54	86	198	**49**	69
	F	**47**	59	81	158	61	78

Table S4. Predictive MAE of energy (meV) and forces (meV/A) on the ground-state azobenzene in xxMD-CASSCF dataset using various loss weights and default MACE model.

	Testing		Validation	
**Loss E:F ratio**	**E**	**F**	**E**	**F**
1000:1	325	210	291	186
100:1	311	104	266	87
10:1	338	72	327	58
1:1	446	66	458	53
1:10	516	64	524	50
1:100	541	65	544	50
1:1000	527	63	527	50

G.1 Dynamics

Initial conformations are generated by Wigner-Sampling of the optimized ground-state structure with the same level of electronic structure method. For each conformation, a single-point calculation is performed to acquire the energy of states without spin-orbit calculations. To select initial excited-states, the MCH representation of the Hamiltonian is used to simulate delta-pulse excitation based on excitation energies and oscillators strengths with an excitation window of 0.0 to 10.0 eV.

For azobenzene, we conducted 300 fs SHARC dynamics simulations with a time step of 0.5 fs. For dithiophene, we conducted 500 fs SHARC dynamics simulations with a time step of 0.5 fs. For malonaldehyde, we conducted 300 fs SHARC dynamics with a timestep of 0.25 fs. For stilbene, we performed 500 fs SHARC dynamics with a time step of 0.5 fs. Local diabatizatrion scheme was used to calculate the non-adiabatic coupling vectors by calculating the overlap matrix of wavefunctions between steps. Non-adiabatic coupling vectors are included in the gradient transformation. kinetic energy are adjusted by rescaling the velocity vectors during a surface hop. When the surface hop is refused due to insufficient energy, the velocity doesn't reflect at a frustrated hop. Default energy-based decoherence scheme was used for decoherence correction. The standard SHARC surface hopping probabilities was used as the surface hopping scheme. All gradients and non-adiabatic couplings of active states were calculated at each time step. For azobenzene, dithiophene, malonaldehyde, and stilbene the threshold of total energy was set to 0.6 eV, 0.2 eV, 0.3 eV and 0.2 eV.

Following is an example input for SHARC dynamics:

```
printlevel 2
geomfile "geom"
veloc external
velocfile "veloc"

nstates 3 0 0
actstates 3 0 0
state 2 mch
coeff auto
```

Table S5. Summary of the computational methods, number of samples used in direct non-adiabatic dynamics sampling for four molecules, and number of data points for all studied molecules. The number in the parenthesis indicates the number of active electrons and orbitals. The total energy conservation (Total E. Con.) criteria has a unit of eV.

Molecule	Method	Total E. Con.	Num. of Samples
Dithiophene	SA-CASSCF(10e,10o)/6-31g	0.2	24769
Azobenzene	SA-CASSCF(6e,6o)/6-31g	0.6	8414
Malonaldehyde	SA-CASSCF(8e,6o)/6-31g	0.3	25568
Stilbene	SA-CASSCF(2e,2o)/6-31g*	0.2	27965

Table S6. Summary of the number of samples used in direct non-adiabatic dynamics sampling for four molecules, and number of data points for all studied molecules.

Molecule	Num. of Samples	Train	Valid	Test
Dithiophene	24769	12400	6169	6200
Azobenzene	8414	4200	2114	2100
Malonaldehyde	25568	14000	6965	7000
Stilbene	27965	12800	6368	6400

```
rngseed -28624

ezero   -536.9454713000
tmax 500.000000
stepsize 0.500000
nsubsteps 25

surf diagonal
coupling overlap
ekincorrect parallel_vel
reflect_frustrated none
decoherence_scheme edc
decoherence_param 0.1
hopping_procedure sharc
```

G.2 Complete Active Space Self-Consistent Field (CASSCF)

In quantum chemistry, accurately capturing electron correlation—the interaction of electrons relative to one another—is pivotal for an in-depth understanding of a molecule's electronic structure. While standard methods like Hartree-Fock (HF) have their strengths, they can falter in specific scenarios. This is where the CASSCF method becomes instrumental.

Central to CASSCF is the categorization of molecular orbitals into three distinct groups:

1. **Inactive (core) orbitals:** These are fully occupied orbitals, exempted from the correlation treatment.
2. **Active orbitals:** A defined number of electrons within these orbitals undergo correlation across a predetermined set of orbitals. The flexibility in electron configuration within the active space encapsulates static electron correlation.
3. **Virtual (secondary) orbitals:** Remaining unoccupied, these orbitals are sidelined from the primary correlation procedure.

The CASSCF methodology initially optimizes the active space orbitals employing a comprehensive configuration interaction (CI) calculation. This act of considering all plausible electron configurations within the active ambit captures static correlation. To address dynamic correlation, supplementary methods, like multi-reference perturbation theory (MRPT), are often invoked.

Advantages of CASSCF:

- Offers a harmonized treatment of electron correlation.

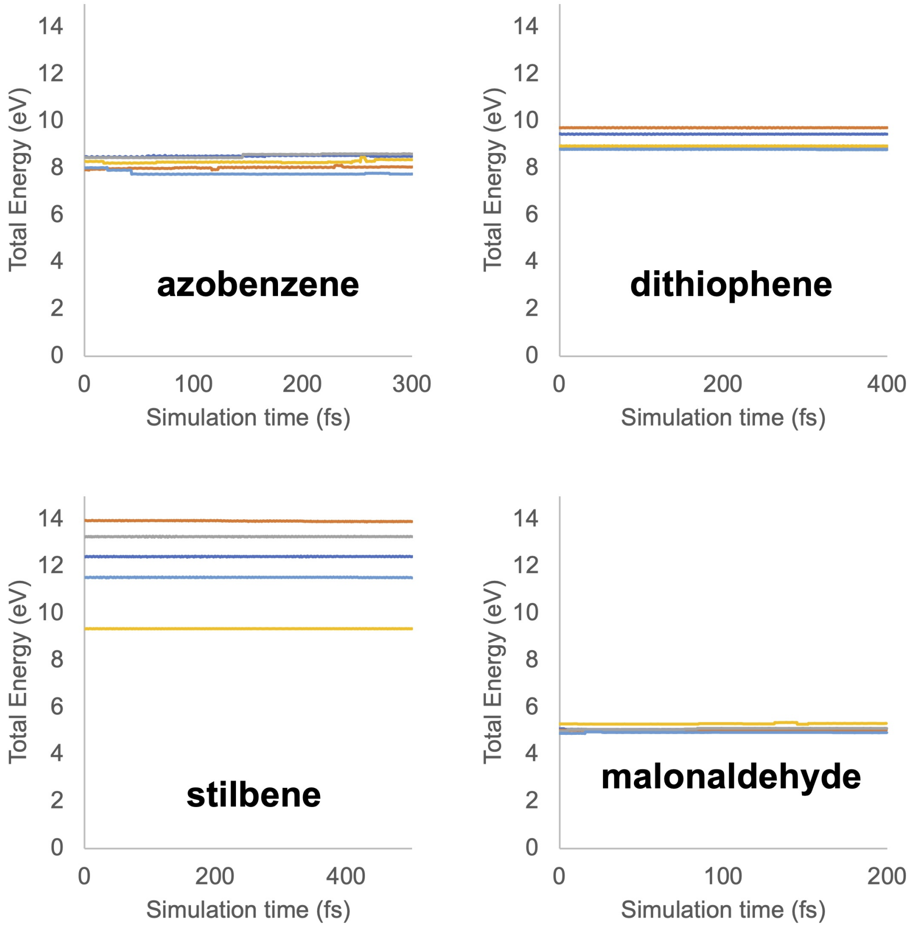

Figure S5. Illustration of total energy conservation over the simulation time of trajectories in xxMD-CASSCF datasets. All trajectories follow the total energy conservation threshold.

- Particularly apt for systems with closely-spaced electronic states, encompassing transition states, metal complexes, and excited states.

However, one should note the substantial computational demands, especially with enlarging active spaces, which can potentially restrict its application or mandate approximate solutions.

For SA-CASSCF calculations, OpenMolcas 22.06 was used, which is available at `https://gitlab.com/Molcas/OpenMolcas`. The active space orbitals of the starting configurations are listed as following (Figure S6).

G.3 Unrestricted KS-DFT

In molecular modeling, the precise representation of electronic configurations during chemical reactions is paramount. The popular restricted KS-DFT inherently pairs electrons, enforcing identical spatial orbitals for both spin-up and spin-down states.

Consider the paradigmatic dissociation of hydrogen (H_2) into atomic hydrogen:

$$H_2 \rightarrow 2H \tag{4}$$

Within the confines of restricted KS-DFT, as H_2 dissociates, the emerging electrons—now localized on individual atoms—are still bound to identical spatial distributions. This treatment may distort the real physical scenario.

Unrestricted KS-DFT, on the other hand, permits differentiation between spin-up and spin-down spatial orbitals, enabling a nuanced portrayal of the process. In the H_2 example, would independently model the electron on each hydrogen atom, providing a truer representation of the physical system.

For all unrestricted KS-DFT calculations, we used M06[9] meta-GGA hybrid functional with 6-31g basis set. All calculations are done with the Psi4[10] package (available at `https://github.com/psi4/psi4`) interfaced the ASE[11] package (available at `https://github.com/rosswhitfield/ase`).

G.4 Dihydrogen dissociation: a comparative case of RKS, UKS and CASSCF

The limitation of using DFT, espeicially restricted DFT becomes evident when examining the H-H bond-breaking process, as illustrated in Figure S7. Here, spin-unpolarized DFT yields an inaccurate yet smooth curve when juxtaposed against its spin-polarized counterpart, with CASSCF serving as the reference.

References

1. Schütt, K. *et al.* Schnet: A continuous-filter convolutional neural network for modeling quantum interactions. *Adv. neural information processing systems* **30** (2017).
2. Gasteiger, J., Giri, S., Margraf, J. T. & Günnemann, S. Fast and uncertainty-aware directional message passing for non-equilibrium molecules. *arXiv preprint arXiv:2011.14115* (2020).
3. Liu, Y. *et al.* Spherical message passing for 3d graph networks. *arXiv preprint arXiv:2102.05013* (2021).
4. Batzner, S. *et al.* E (3)-equivariant graph neural networks for data-efficient and accurate interatomic potentials. *Nat. communications* **13**, 2453 (2022).
5. Musaelian, A. *et al.* Learning local equivariant representations for large-scale atomistic dynamics. *Nat. Commun.* **14**, 579 (2023).
6. Batatia, I., Kovacs, D. P., Simm, G., Ortner, C. & Csányi, G. Mace: Higher order equivariant message passing neural networks for fast and accurate force fields. *Adv. Neural Inf. Process. Syst.* **35**, 11423–11436 (2022).
7. McQuarrie, D. A. & Simon, J. D. *Molecular thermodynamics* (University Science Books, 1999).
8. Stocker, S., Gasteiger, J., Becker, F., Günnemann, S. & Margraf, J. T. How robust are modern graph neural network potentials in long and hot molecular dynamics simulations? *Mach. Learn. Sci. Technol.* **3**, 045010 (2022).
9. Zhao, Y. & Truhlar, D. G. The m06 suite of density functionals for main group thermochemistry, thermochemical kinetics, noncovalent interactions, excited states, and transition elements: two new functionals and systematic testing of four m06-class functionals and 12 other functionals. *Theor. chemistry accounts* **120**, 215–241 (2008).
10. Turney, J. M. *et al.* Psi4: an open-source ab initio electronic structure program. *Wiley Interdiscip. Rev. Comput. Mol. Sci.* **2**, 556–565 (2012).
11. Larsen, A. H. *et al.* The atomic simulation environment—a python library for working with atoms. *J. Physics: Condens. Matter* **29**, 273002 (2017).

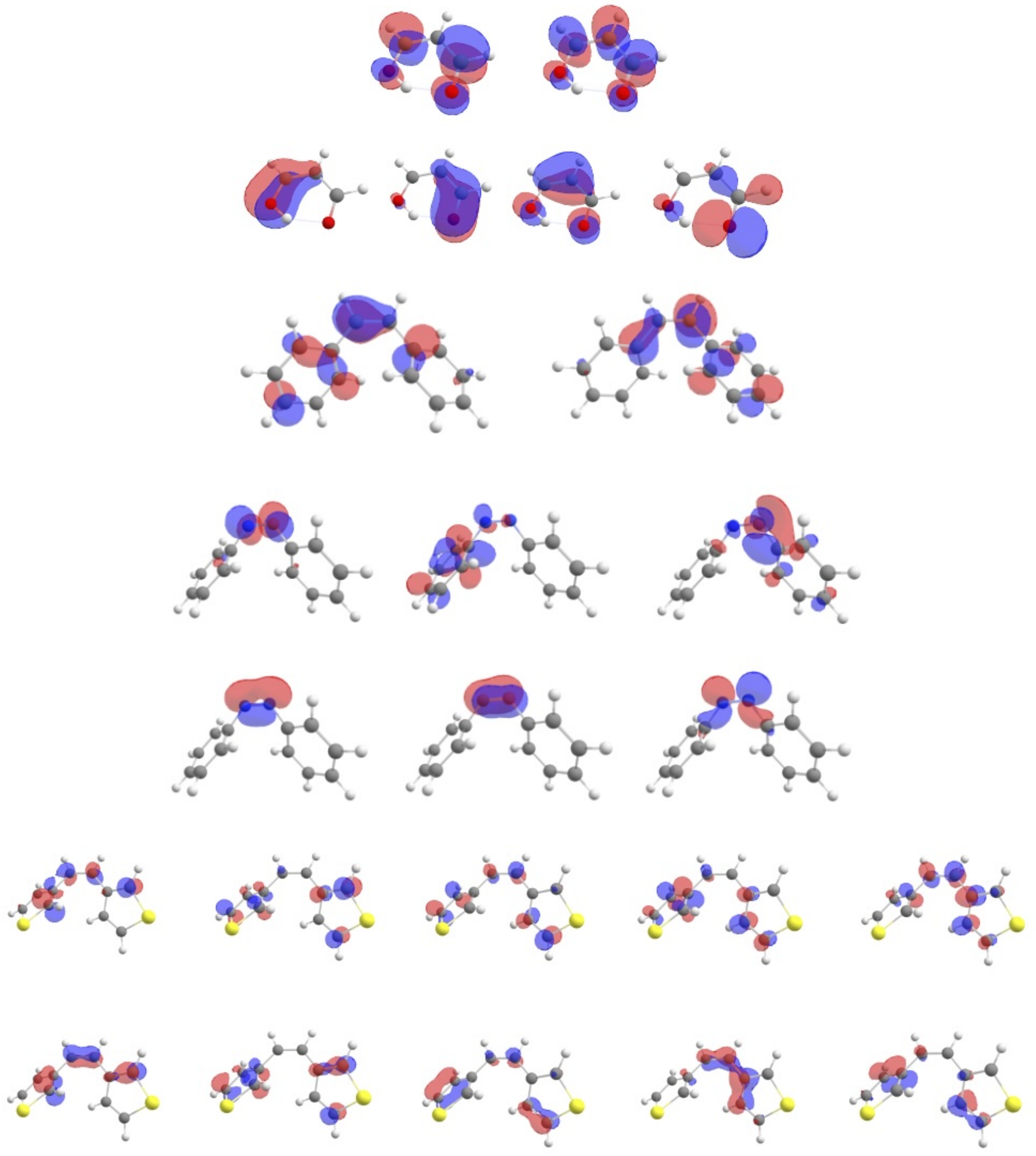

Figure S6. Active space orbitals used SA-CASSCF calculations for malonaldehyde, stilbene, azobenzene and dithiophine.

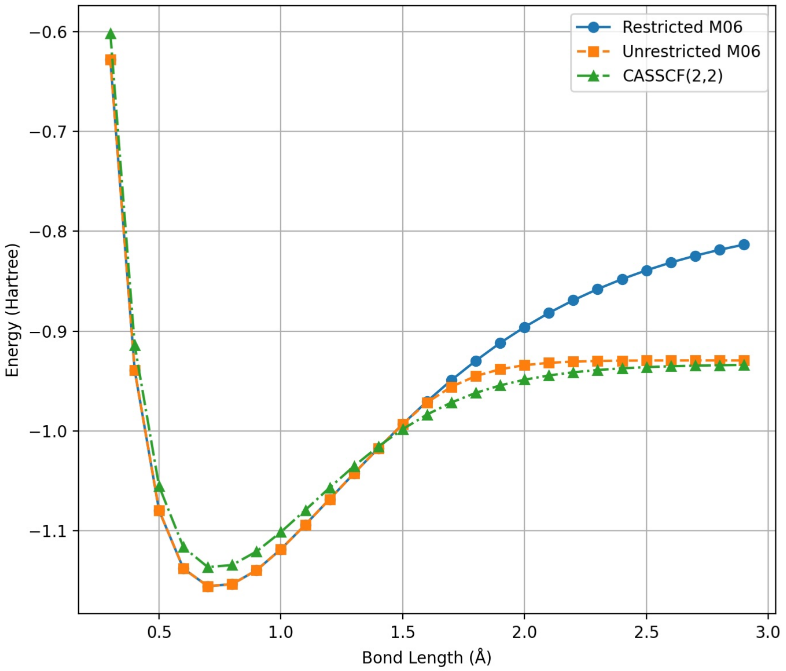

Figure S7. Dissociation Curve of dihydrogen molecule using RKS, UKS, and CASSCF(2,2) methods. RKS is inherently inadequate for capturing the true electronic structure nuances of bond-breaking events, as seen in the deviation from the CASSCF. In principle, multi-reference methods are essential for accurate modeling of such chemical reactions, ensuring a more holistic representation of the electronic correlation effects.
